# Staufen1 Interacts with Multiple Components of the Ebola Virus Ribonucleoprotein and Enhances Viral RNA Synthesis

**DOI:** 10.1128/mBio.01771-18

**Published:** 2018-10-09

**Authors:** Jingru Fang, Colette Pietzsch, Palaniappan Ramanathan, Rodrigo I. Santos, Philipp A. Ilinykh, Mariano A. Garcia-Blanco, Alexander Bukreyev, Shelton S. Bradrick

**Affiliations:** a Department of Biochemistry & Molecular Biology, University of Texas Medical Branch, Galveston, Texas, USA; b Department of Pathology, University of Texas Medical Branch, Galveston, Texas, USA; c Galveston National Laboratory, University of Texas Medical Branch, Galveston, Texas, USA; d Programme in Emerging Infectious Diseases, Duke-NUS Medical School, Singapore, Singapore; e Department of Microbiology & Immunology, University of Texas Medical Branch, Galveston, Texas, USA; St. Jude Children's Research Hospital; Colorado State University; National Institute of Allergy and Infectious Diseases, National Institutes of Health

**Keywords:** RNA binding proteins, RNA replication, ebola virus, virus-host interactions

## Abstract

Ebola virus (EBOV) genome and mRNAs contain long, structured regions that could hijack host RNA-binding proteins to facilitate infection. We performed RNA affinity chromatography coupled with mass spectrometry to identify host proteins that bind to EBOV RNAs and identified four high-confidence proviral host factors, including Staufen1 (STAU1), which specifically binds both 3′ and 5′ extracistronic regions of the EBOV genome. We confirmed that EBOV infection rate and production of infectious particles were significantly reduced in STAU1-depleted cells. STAU1 was recruited to sites of EBOV RNA synthesis upon infection and enhanced viral RNA synthesis. Furthermore, STAU1 interacts with EBOV nucleoprotein (NP), virion protein 30 (VP30), and VP35; the latter two bridge the viral polymerase to the NP-coated genome, forming the viral ribonucleoprotein (RNP) complex. Our data indicate that STAU1 plays a critical role in EBOV replication by coordinating interactions between the viral genome and RNA synthesis machinery.

## Abstract (precis)

Ebola virus (EBOV) is a negative-strand RNA virus with significant public health importance. Currently, no therapeutics are available for Ebola, which imposes an urgent need for a better understanding of EBOV biology. Here we dissected the virus-host interplay between EBOV and host RNA-binding proteins. We identified novel EBOV host factors, including Staufen1, which interacts with multiple viral factors and is required for efficient viral RNA synthesis.

## INTRODUCTION

Ebola virus (EBOV) has caused multiple human outbreaks, notably the 2013–2016 catastrophic epidemic in West Africa which led to more than 11,000 fatalities (1–3), and a new outbreak recently occurred in The Democratic Republic of the Congo (4). At present, no FDA-approved treatment is available for EBOV infections. Due in part to the high case fatality rate of up to 90% observed in infected patients, EBOV has been classified as a category A pathogen, posing a high risk to public health (5).

EBOV belongs to the family *Filoviridae* in the order *Mononegavirales* (6) and features a nonsegmented, negative-sense (NNS) RNA genome. The RNA genome is tightly encapsidated by viral nucleoprotein (NP) oligomers and closely associated with the viral RNA synthesis machinery, which is composed of the viral RNA-dependent RNA polymerase (L), the polymerase cofactor virion protein 35 (VP35), and the transcription activator VP30 (7). Together, they form a multiprotein-RNA complex, termed the viral ribonucleoprotein (RNP) complex (8). The NP-coated RNA genome serves as the template for synthesis of seven monocistronic, capped and polyadenylated mRNAs. In addition, the genome is the template for synthesis of the replicative intermediate, or antigenome (9), which in turn serves as the template for synthesis of progeny genomes (10, 11). EBOV must have evolved a comprehensive strategy to regulate its RNA synthesis, coordinating viral mRNA transcription, genome replication, and viral RNP assembly; however, host factors participating in these processes are poorly understood.

The EBOV genome contains regions with predicted stable RNA structures (12). The genome contains terminal extracistronic regions, termed the 3′ leader and 5′ trailer, that are essential for RNA synthesis (13–15), and many of the viral transcripts have long untranslated regions (UTRs) (16). To some extent, the functional requirement of extracistronic regions for EBOV RNA synthesis has been documented by minigenome assays (17). For example, an important *cis*-acting RNA structure required for EBOV transcriptional regulation is located in the 3′ extracistronic region of the viral genome and functions through interaction with the viral *trans*-acting factor, VP30 (18–21). Nevertheless, functionality of most EBOV RNA secondary structures and potential host or viral *trans*-acting factors that recognize these structures have not been well established (22, 23).

Many RNA viruses induce the formation of discrete intracellular compartments that sequester viral RNA synthesis (24, 25). Previously published studies have implicated distinct EBOV-induced intracellular compartments, termed inclusion bodies (26), as the sites of EBOV RNA synthesis and generation of progeny viral RNPs (27–29). In contrast to viral proteins and RNA, few cellular factors, including several proteins implicated in translation such as eukaryotic initiation factor 4G (eIF4G), eIF3B, and poly(A)-binding protein, have been shown to localize to inclusion bodies. The functional outcome of redistribution of these cellular factors into EBOV inclusion body remains unclear (30).

While the EBOV genome is prominently coated with NP in infected cells, we posit that highly structured terminal regions of the genome may be available for interaction with other cellular and viral proteins, such as VP30. To identify host RNA-binding proteins (RBPs) that interact with the long and highly structured EBOV RNAs, we used RNA affinity chromatography combined with mass spectrometry (RAC-MS) and analyzed the functional effects of candidate EBOV RBPs on viral infection. We focused on Staufen1 (STAU1), which preferentially associates with both 3′ and 5′ extracistronic regions of EBOV genomic RNA, as an important EBOV proviral host factor. We demonstrated that STAU1 enhances EBOV RNA synthesis and that STAU1 is recruited to EBOV inclusion bodies during viral infection. Unexpectedly, we also revealed that endogenous STAU1 associates with multiple components of the EBOV RNP. Our observations suggest STAU1 is a cellular constituent of EBOV inclusion bodies that is actively engaged in the process of EBOV RNA synthesis.

## RESULTS

### Identification of cellular factors that bind to EBOV RNA and regulate viral infection.

**FIG 1:**
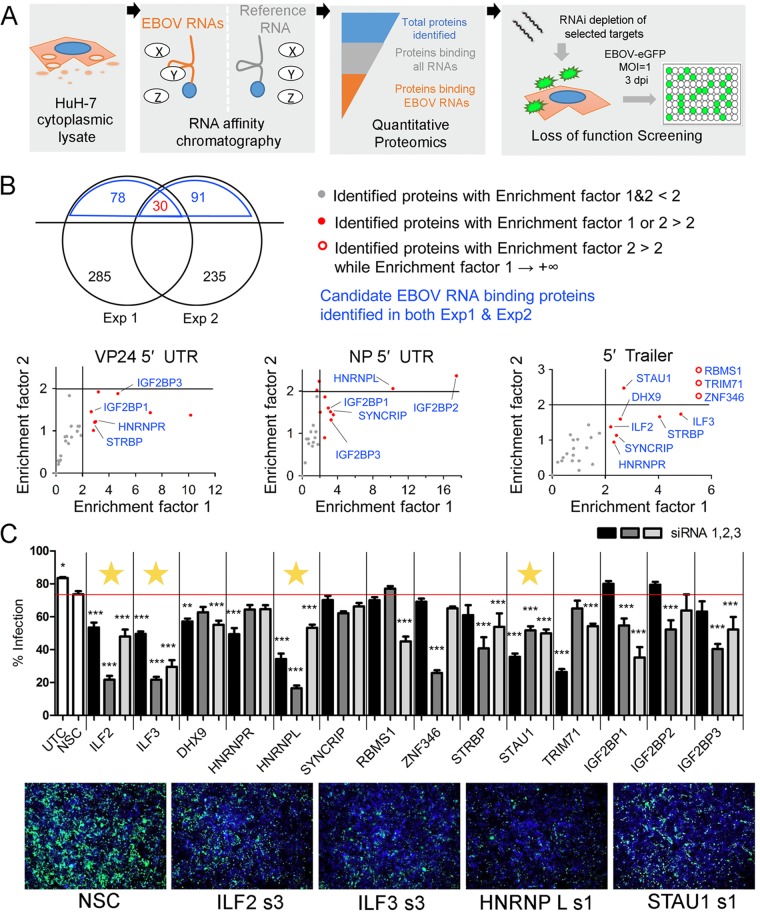
Identification of cellular factors that bind to EBOV RNA and regulate viral infection. (A) Workflow of RAC-MS and loss-of-function screening. Candidate cellular RBPs interacting with EBOV RNA were identified by two independent RAC-MS experiments. *In vitro*-transcribed EBOV 5′ trailer, NP mRNA 5′ UTR, and VP24 mRNA 5′ UTR were used as EBOV RNA baits; cellular DDX39B mRNA 5′ UTR was used as the reference RNA bait. Immobilized RNAs or matrix alone was incubated with HuH-7 cell lysate, and isolated proteins were analyzed by quantitative mass spectrometry. A total of 14 proteins identified as enriched for EBOV RNAs across two experiments were screened in infection assays. RNAi, RNA interference; dpi, days postinfection. (B) EBOV RBPs identified by RAC-MS. (Top) Total numbers of proteins identified in each experiment are shown in black. Numbers of proteins enriched two-fold or greater for any of the RNA baits compared to that for the matrix control are depicted in blue, and the number of proteins shared in the two experiments is shown in red. Exp, experiment. (Bottom) EBOV RBPs identified by RAC-MS. The 30 proteins common to both experiments were analyzed by two criteria in order to identify factors enriched for EBOV RNAs. Enrichment factor 1 is the abundance ratio for proteins binding the indicated EBOV RNA construct compared to the reference RNA. Enrichment factor 2 is defined as the abundance ratio for proteins binding the indicated EBOV RNA compared to the average for all RNA baits. Proteins with enrichment factor 1 or 2 of >2 were compared across two independent experiments are shown in blue. See also Fig. S2 in the supplemental material. (C) (Top) Loss-of-function screen to evaluate the importance of identified EBOV RBPs. Candidate EBOV RBPs were depleted by three distinct siRNAs in HuH-7 cells that were subsequently infected with an EBOV-eGFP recombinant virus at an MOI of 1 PFU per cell. Cells were analyzed for rate of infection at 3 days p.i. Negative-control siRNA (NSC) and untreated control (UTC) are indicated. Results from two independent experiments were analyzed using one-way analysis of variance (ANOVA) with Bonferroni’s multiple-comparison test. Values are means plus standard error of the means (error bars). Values that are significantly different from the value for the NSC are indicated by asterisks as follows: *, *P*  < 0.05; **, *P*  < 0.005; ***, *P*  < 0.0005. Proteins for which all three siRNAs significantly altered infection are indicated with a yellow star. (Bottom) Representative fluorescent images of HuH-7 monolayers at 3 days postinfection showing the GFP-positive cells (green) and nuclei (blue) for negative control and selected target knockdown cells.

We hypothesized that certain EBOV RNAs predicted to form stable secondary structures could serve as *cis*-acting elements to hijack cellular factors that facilitate viral infection. To begin testing this hypothesis, we performed an RNA affinity chromatography combined with mass spectrometry (RAC-MS) screen to identify host proteins interacting with selected EBOV RNA regions (Fig. 1A). We chose a RAC method that has successfully been used to identify host RNA-binding proteins (RBPs) that bind to UTRs of dengue virus genomes and takes advantage of the tobramycin RNA aptamer that allows reversible coupling of RNA transcripts to a tobramycin affinity matrix (31–34). Previous work describing functional roles for EBOV 5′ UTRs in translational regulation was inspired by the fact that EBOV UTR sequences are, on average, longer than those of other nonsegmented, negative-sense RNA viruses (35). On the basis of a similar rationale, we selected the two longest EBOV mRNA 5′ UTRs that are predicted to have extensive RNA secondary structure (see Fig. S1 in the supplemental material): the 5′ UTR of NP mRNA (414 nucleotides [nt]) and the 5′ UTR of VP24 mRNA (460 nt). In addition, we analyzed the 5′ trailer of genomic RNA (676 nt), another long, structured extracistronic region. For comparison, the 5′ UTR of host mRNA from the DDX39B gene (502 nt) was chosen as reference RNA. Human HuH-7 hepatoma cell lysate was used for identification of EBOV RNA-binding proteins, as liver is one of the main target organs of EBOV (36). Proteins identified across two independent experiments are shown in Data Set S1 in the supplemental material.

10.1128/mBio.01771-18.1 FIG S1 Predicted secondary structure of EBOV RNAs used in RAC. Minimum free energy structure of EBOV noncoding RNAs predicted by RNAfold 2.4.6. based on the primary sequence of each RNA. RNA structures are colored by base-pairing probabilities. For unpaired regions, the color denotes the probability of being unpaired. (A) EBOV genome 5′ trailer. (B) EBOV genome 3′ extracistronic region. (C) EBOV VP24 mRNA 5′ UTR. (D) EBOV NP mRNA 5′ UTR. Download FIG S1, TIF file, 15.7 MB. Copyright © 2018 Fang et al. 2018 Fang et al. This content is distributed under the terms of the Creative Commons Attribution 4.0 International license.

10.1128/mBio.01771-18.10 DATA SET S1 Summary of host RNA-binding proteins (RBPs) identified by RAC-MS. Results from two independent RAC-MS experiments are shown. Download Data Set S1, XLSX file, 0.0 MB. Copyright © 2018 Fang et al. 2018 Fang et al. This content is distributed under the terms of the Creative Commons Attribution 4.0 International license.

10.1128/mBio.01771-18.2 FIG S2 Scatter plots of a repeat experiment showing that host RBPs associate with EBOV RNA identified by RAC-MS. Thirty protein candidates selected in the main article were further analyzed by two additional criteria in order to identify proteins that interact with EBOV RNA. We arbitrarily defined the ratio of normalized total spectra (NTS) for a given EBOV RNA construct to that of the reference RNA construct as enrichment factor 1. Likewise, the ratio of NTS of a particular EBOV RNA construct to the average NTS of all RNA constructs was arbitrarily defined as enrichment factor 2. Identified proteins with an enrichment factor 1 or 2 of >2 were compared across two independent experiments. Proteins that met the criteria described above in both experiments are shown in blue. Download FIG S2, TIF file, 26.5 MB. Copyright © 2018 Fang et al. 2018 Fang et al. This content is distributed under the terms of the Creative Commons Attribution 4.0 International license.

We used multiple criteria to classify proteins identified by quantitative MS. The first criterion excluded proteins that nonspecifically bound to the matrix. After ruling out nonspecific interactors, we identified 30 host RBPs shared in both experiments (Fig. 1B). We then classified proteins that bound to one or more EBOV RNAs with higher abundance (twofold or more) than to the DDX39B 5′ UTR. This identified 14 factors that were consistently enriched in both experiments as candidate EBOV RBPs (Fig. 1B and Fig. S2). As expected, the majority of these proteins have been previously reported as RBPs (37–44). Interleukin enhancer-binding factor 2 (ILF2), interleukin enhancer-binding factor 3 (ILF3), DExH-box helicase 9 (DHX9), RNA binding motif single-stranded interacting protein 1 (RBMS1), zinc finger protein 346 (ZNF346), Staufen double-stranded RNA-binding protein 1 (Staufen1 [STAU1]), and tripartite motif containing 71 (TRIM71) demonstrated selective binding to the 5′ trailer of EBOV genomic RNA. On the other hand, insulin-like growth factor 2 mRNA-binding proteins 1 and 3 (IGF2BP1 and IGF2BP3) demonstrated selective binding to both EBOV 5′ UTRs (Fig. 1B and Fig. S2).

We next asked whether the 14 hits identified by RAC-MS affect EBOV infection of HuH-7 cells. We performed small interfering RNA (siRNA)-mediated depletion of each target protein, followed by infection with a replication-competent recombinant EBOV expressing enhanced green fluorescent protein (eGFP) (EBOV-eGFP) (45), and measured the percentages of cells infected. Cells were transfected with either a nonsilencing control siRNA (NSC) or three independent siRNAs (S1/2/3) targeting the mRNA for each of the selected proteins and infected with EBOV-eGFP at a multiplicity of infection (MOI) of 1 PFU per cell. In general, the majority of siRNAs tested resulted in decreases in the rate of infection. Among the 14 hits, depletion of ILF2, ILF3, heterogeneous nuclear ribonucleoprotein L (HNRNPL), and STAU1 significantly decreased EBOV-eGFP infection for each siRNA used (Fig. 1C). For ILF2, ILF3, and HNRNPL, some siRNAs moderately reduced cell numbers, reflecting lower cell proliferation or a degree of toxicity that may influence EBOV infection (Fig. S3). In contrast, none of the three siRNAs targeting STAU1 reduced cell numbers. Together, these data suggest that these four proteins represent host factors required for efficient replication of EBOV in HuH-7 cells.

10.1128/mBio.01771-18.3 FIG S3 Effects of siRNA transfection on cell numbers in the mini-siRNA screen. Cell numbers for siRNAs were normalized to the value for the nonsilencing control (NSC). Values are means and standard errors of the means. Values that are significantly different are indicated by asterisks as follows: *, *P* < 0.05; **, *P* < 0.005; ***, *P* < 0.0005. Download FIG S3, TIF file, 15.3 MB. Copyright © 2018 Fang et al. 2018 Fang et al. This content is distributed under the terms of the Creative Commons Attribution 4.0 International license.

### STAU1 is an EBOV proviral host factor that binds to both 3′ and 5′ extracistronic regions of the EBOV genome.

**FIG 2:**
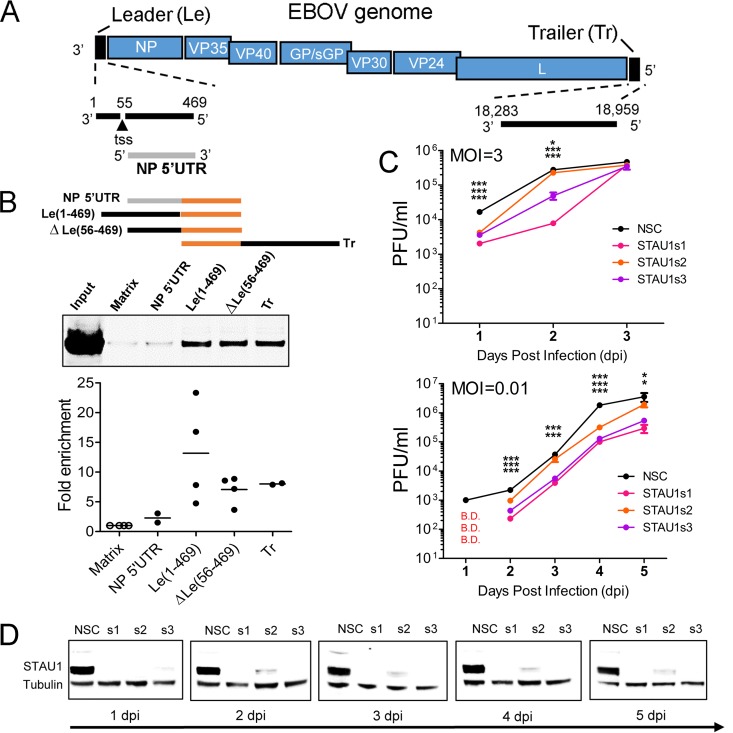
STAU1 is an EBOV proviral host factor and binds to 3′ and 5′ extracistronic regions of the viral genome. (A) Schematic of EBOV RNAs used in RAC for Western blot (WB) analysis. *In vitro*-transcribed EBOV NP 5′ UTR, 3′ extracistronic region [Le(1-469)], truncated 3′ extracistronic region [ΔLe(56-469)], and 5′ trailer (Tr) were used as RNA baits in RAC-WB experiments with HuH-7 cell lysate. Matrix alone served as a negative control. (B) Selective association of the 3′ and 5′ extracistronic regions of the EBOV genome with endogenous STAU1. Proteins pulled down were analyzed by WB performed with anti-STAU1 antibody. A representative blot is shown. Quantitation of bands corresponding to STAU1 from two independent experiments is shown (range and mean values, with the value for matrix normalized to 1). (C) Effect of STAU1 knockdown on EBOV growth kinetics. HuH-7 cells were transfected with STAU1 or nonsilencing control (NSC) siRNAs 48 h prior to infection with EBOV-eGFP at an MOI of 3 PFU per cell (one-step growth kinetics) or at an MOI of 0.01 PFU per cell (multistep growth kinetics). Supernatants were collected at the indicated time points, and infectious particle production was determined by plaque assay. B.D., below detection. Values are means ± standard errors of the means (error bars). Values that are significantly different by unpaired, two-tailed *t* test are indicated by asterisks as follows: *, *P*  < 0.05; ***, *P*  < 0.0005. (D) Western blot demonstrating kinetics of STAU1 knockdown in HuH-7 cells corresponding to indicated time points in EBOV growth kinetics.

We next focused on the role of the double-stranded RNA-binding protein STAU1, because it has not been previously implicated as an EBOV host factor, but it has been reported to play important roles for other RNA viruses (46–48). First, we sought to validate the interaction between STAU1 and EBOV RNA. We repeated RAC assays and detected STAU1 by Western blotting. In addition to the 5′ trailer RNA used for RAC-MS, we also analyzed RNAs encompassing the 3′ extracistronic region of the EBOV genome with the 55-nt 3′ leader sequence Le(1-469) or without the 55-nt 3′ leader sequence ΔLe(56-469) (Fig. 2A and B). Both the 3′ extracistronic region and 5′ trailer of EBOV contain essential signals required for EBOV RNA synthesis (13). We observed that endogenous STAU1 bound the EBOV 5′ trailer RNA more strongly than the 5′ UTR of NP mRNA, validating the RAC-MS data. Interestingly, in addition to the EBOV 5′ trailer, we also found that STAU1 bound to the EBOV 3′ extracistronic region (positions 56 to 469) independently of the 55-nt leader sequence (Fig. 2B). To extend these observations, we performed RNA immunoprecipitation (IP) from 293T cells transiently expressing EBOV minigenome RNA (see below) with or without overexpression of Flag-tagged STAU1 (STAU1-Flag). IP was performed, and RNA coprecipitating with STAU1-Flag or negative-control beads was subjected to reverse transcription-quantitative PCR (RT-qPCR) for measurements of EBOV minigenome RNA and 18S rRNA. This analysis revealed that STAU1-Flag IP enriched EBOV minigenome RNA by approximately sixfold compared to the negative control, whereas 18S rRNA levels were similar for both (Fig. S4). Together, these data indicate that STAU1 preferentially interacts with the terminal extracistronic regions of the EBOV genome *in vitro*.

10.1128/mBio.01771-18.4 FIG S4 RNA immunoprecipitation of EBOV minigenome RNA with STAU1-Flag. 293T cells were transfected with the EBOV minigenome plasmid, a T7 RNA polymerase expression plasmid, and either vector or STAU1-Flag expression plasmid. RNA-IP was performed with Flag antibody, and 18S rRNA/EBOV minigenome RNAs were quantified by RT-qPCR. For each RNA, the negative control (IP from vector-transfected cells) is normalized to one. The results of two independent experiments are shown. Download FIG S4, TIF file, 9.5 MB. Copyright © 2018 Fang et al. 2018 Fang et al. This content is distributed under the terms of the Creative Commons Attribution 4.0 International license.

To confirm the importance of STAU1 for EBOV infection, we characterized the growth kinetics of EBOV-eGFP in STAU1-depleted HuH-7 cells in both one-step (MOI of 3 PFU per cell) and multistep (MOI of 0.01 PFU per cell) infection assays. We performed siRNA-mediated depletion of STAU1 in HuH-7 cells, followed by infection of EBOV-eGFP, and determined the yield of infectious particles secreted into cell supernatants by plaque assay. We also analyzed the kinetics of STAU1 depletion by individual siRNAs. For the one-step growth curve experiment, the yield of infectious particles in STAU1-depleted cells was significantly lower at days 1 and 2 postinfection than in cells transfected with NSC siRNA, indicating that STAU1 knockdown significantly slows the kinetics of EBOV infection (Fig. 2C). For the multistep growth curve experiment, most siRNA/time point combinations showed reduced yield of infectious particles in STAU1-depleted cells on days 2 to 5 postinfection (Fig. 2C). Furthermore, two STAU1 siRNAs (s1 and s3) showed stronger STAU1 depletion, and this was correlated with larger effects on the yield of infectious virus compared to the s2 siRNA (Fig. 2D). In summary, these data show that STAU1 promotes efficient infection of HuH-7 cells by EBOV.

### STAU1 is recruited to EBOV inclusion bodies.

Given that STAU1 was identified as a putative EBOV RBP, we hypothesized that it may be in close proximity to EBOV inclusion bodies where EBOV RNA synthesis takes place (27). To determine whether STAU1 is recruited to EBOV inclusion bodies upon viral infection, we performed a time course infection and visualized localization of endogenous STAU1 by immunofluorescence analysis (IFA). It has been previously shown that EBOV VP35 functions as a component of viral RNA synthesis machinery (49) and localizes to EBOV inclusion bodies (28). Therefore, we used it as a marker for EBOV inclusion bodies in infected cells.

**FIG 3:**
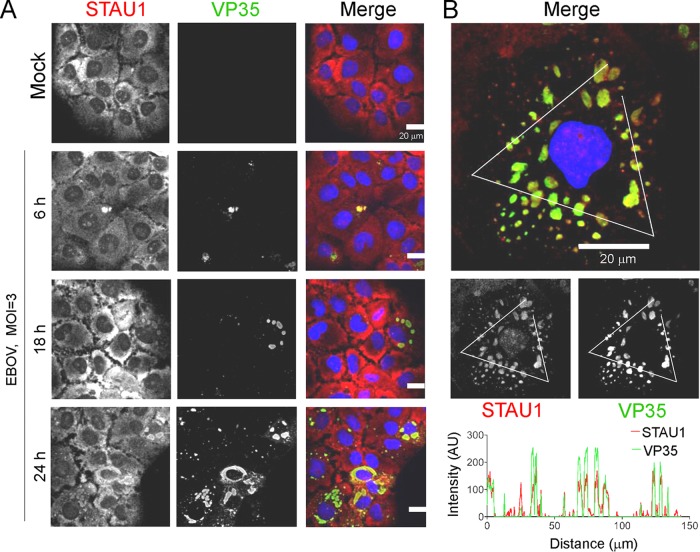
STAU1 is recruited to viral inclusion bodies upon EBOV infection. (A) HuH-7 cells were transfected with nonsilencing control siRNA 48 h prior to infection with EBOV at an MOI of 3 PFU per cell. Cells were fixed at 6, 18, and 24 h postinfection and immunostained with anti-STAU1 antibodies (red) and anti-EBOV VP35 antibodies (green) for confocal microscopy. Nuclei were visualized by Hoechst staining. Representative fields are shown from two independent experiments with duplicate wells. Bars = 20 μm. (B) Colocalization analysis of endogenous STAU1- and VP35-containing EBOV at 24 h postinfection (MOI of 3 PFU per cell) was conducted along the white segmented line for each channel. The line profile plot of the fluorescence intensity (in arbitrary units [AU]) of STAU1 and EBOV VP35 in the same focal plane is shown.

In mock-infected HuH-7 cells, STAU1 was observed to localize diffusely in the cytoplasm, which is consistent with previous reports describing STAU1 localization to the rough endoplasmic reticulum (Fig. 3A) (50). At 6 h postinfection (hpi), only a minority of cells had VP35 signal, which was distributed in an amorphous fashion, while the overall organization of STAU1 did not show any significant change in pattern. At 18 hpi, changes in STAU1 localization started to become apparent: small spherical EBOV inclusion bodies started to form in the cytosol in a few cells, and a portion of endogenous STAU1 colocalized with these EBOV inclusion bodies. At 24 hpi, most cells were VP35 positive with sizable EBOV inclusion bodies that colocalized with STAU1 in most cells (Fig. 3A). We further analyzed STAU1-VP35 colocalization in HuH-7 cells at 24 hpi by line profile analysis and found highly correlated distribution of STAU1 and VP35 signals (Fig. 3B).

We next sought to determine whether STAU1 is important for the formation of EBOV inclusion bodies. Analysis of the quantity of EBOV inclusion bodies revealed no differences in STAU1-depleted cells compared to control cells (Fig. S5A and B), suggesting that STAU1 is dispensable for EBOV inclusion body formation. Despite this, our results showed that endogenous STAU1 still colocalized with EBOV inclusion bodies when its protein level was significantly depleted. These observations suggest a possible role for STAU1 in EBOV RNA synthesis that is thought to take place exclusively within inclusion bodies.

10.1128/mBio.01771-18.5 FIG S5 STAU1 is dispensable for EBOV inclusion body formation. HuH-7 cells were transfected with 10 nM STAU1 siRNA or nonsilencing control siRNA (NSC) 48 hours prior to infection with EBOV at an MOI of 3 PFU per cell. At 24 hours postinfection, cells were fixed and immunostained with anti-STAU1 (red) and anti-eVP35 (green) antibodies for confocal microscopy. Nuclei were visualized by Hoechst staining. (A) Representative fields are shown from two independent experiments with duplicate wells. The number and area of EBOV inclusion bodies per cell were measured in a total of 639 cells. In each experiment, the number and area of EBOV inclusion bodies in STAU1-depleted cells were normalized to the value for the NSC control (set at 100%). (B) Percent EBOV inclusion body count/area per cell was analyzed using unpaired, two-tailed *t* test under each condition compared to the values for controls. The means and standard errors of means are shown. (C) A representative Z-stack analysis of an inset in panel A with 4× magnification of the merged image. Images on the bottom and right side depict the Z-axis. Bar = 20 μm. Download FIG S5, TIF file, 91.2 MB. Copyright © 2018 Fang et al. 2018 Fang et al. This content is distributed under the terms of the Creative Commons Attribution 4.0 International license.

### STAU1 promotes EBOV RNA synthesis.

**FIG 4:**
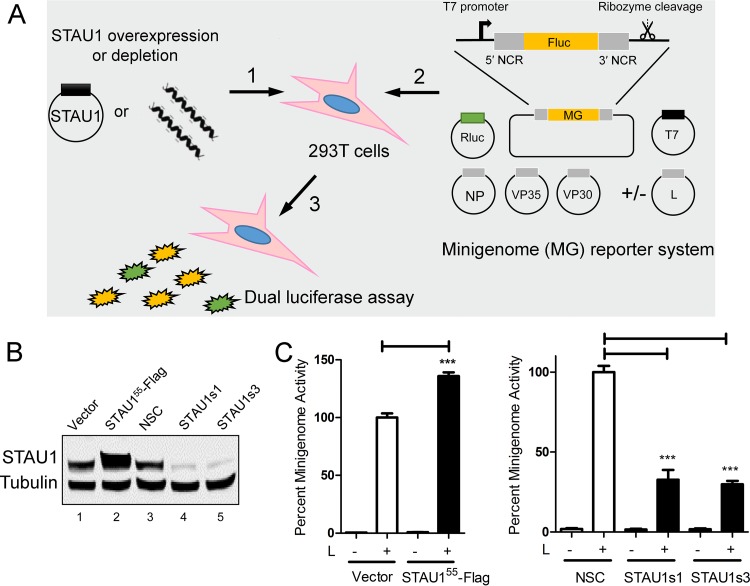
STAU1 promotes EBOV RNA synthesis. (A) Schematic of the EBOV minigenome (MG) reporter assay. Transfection of T7 polymerase expression plasmid and a plasmid encoding Fluc reporter flanked by the 3′ and 5′ extracistronic regions of authentic EBOV genome produces a negative-sense, reporter viral RNA. Once transcribed, the 3′ hepatitis delta virus (HDV) ribozyme autocatalytically cleaves itself off, creating a defined 3′ minigenome termini. EBOV RNP proteins (NP, VP35, VP30, and L) are coexpressed to promote transcription and replication of the minigenome. An Rluc expression plasmid is cotransfected for normalization of transfection efficiency. NCR, noncoding RNA. (B) Representative Western blot from three independent experiments demonstrating STAU1 overexpression or knockdown in 293T cells at the time of replicon plasmid transfection. (C) Role of STAU1 in EBOV minigenome replication. Cells were lysed 48 h posttransfection, and dual-luciferase assay was performed. EBOV minigenome activity in 293T cells was determined by normalizing the Fluc reporter against Rluc signal. In each experiment, EBOV minigenome activities were normalized to the values for controls (set at 100%). Results from three independent experiments with triplicate samples were analyzed by using unpaired, two-tailed *t* test under each condition compared to controls. Values are means plus standard errors of the means. Values that are significantly different (*P*  < 0.0005) by unpaired, two-tailed *t* test are indicated by three asterisks.

We next asked whether STAU1 facilitates viral RNA synthesis. To quantify EBOV RNA synthesis independently from other steps of the viral life cycle, we used a previously established EBOV minigenome system that recapitulates genome transcription, replication, and encapsidation (13) (Fig. 4A). We opted to use 293T cells for these experiments, since we were unable to detect minigenome activity in transfected HuH-7 cells (unpublished data). In the EBOV minigenome plasmid, the entire coding sequence is replaced by a firefly luciferase (Fluc) reporter gene that is flanked by *bona fide* 3′ and 5′ extracistronic regions of the EBOV genome required for EBOV RNA synthesis. Viral proteins (NP, VP35, VP30, and L) are supplied by cotransfection of expression plasmids, and a *Renilla* luciferase (Rluc) expression plasmid is included to normalize for transfection efficiency. The resulting Fluc signal normalized against control Rluc signal is taken as a measure of the activity of EBOV RNA synthesis, which reflects a combination of both genome replication and transcription. We depleted or overexpressed STAU1 in 293T cells and transfected the minigenome system plasmids 48 h later. We observed that cells overexpressing STAU1 (Fig. 4B) showed significantly enhanced EBOV minigenome activity compared to control cells (Fig. 4C, left panel). On the other hand, in STAU1-depleted cells (Fig. 4B), the EBOV minigenome activity was significantly decreased compared to the negative control (Fig. 4C, right panel). Together, these results indicate that STAU1 is required for efficient EBOV RNA synthesis.

### STAU1 interacts with major components of the EBOV RNP.

**FIG 5:**
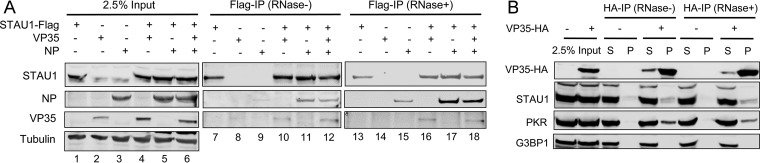
STAU1 interacts with essential components of the EBOV RNP. (A) STAU1 coimmunoprecipitates VP35 and NP in an RNase-resistant manner. Representative Western blot analysis from three independent co-IP experiments performed in 293T cells is shown. (B) VP35 coimmunoprecipitates endogenous STAU1 and PKR in an RNase-resistant manner. Representative Western blot analysis from two independent experiments is shown. S, supernatant; P, pellet.

To further understand the mechanism by which STAU1 enhances EBOV infection, we probed for physical interactions between STAU1 and isolated components of the EBOV RNP. Specifically, we asked whether STAU1 could interact directly or indirectly with VP35 and/or NP in the absence of active virus infection using coimmunoprecipitation (co-IP) assays. 293T cells were cotransfected with plasmids expressing recombinant Flag-tagged STAU1 and either hemagglutinin (HA)-tagged VP35 (HA-VP35) (Fig. 5A, lane 4) or NP (Fig. 5A, lane 5). In addition, cells transfected with plasmids expressing either Flag-STAU1, HA-VP35, or NP alone served as controls (Fig. 5A, lanes 1 to 3). Forty-eight hours posttransfection, anti-Flag IPs were performed using whole-cell lysate in the presence or absence of RNase A/T1 treatment. VP35 and NP were found to co-IP with STAU1 under both conditions, suggesting that the interactions are not bridged by RNA (Fig. 5A, lanes 10, 11, 16, and 17). In the same experiment, cells were cotransfected with plasmids expressing all three proteins (Fig. 5A, lane 6), and STAU1 was found to similarly co-IP both VP35 and NP (Fig. 5A, lanes 12 and 18). Notably, we used a particular NP/VP35 ratio (1:0.25) in transfections based on the results of a previous study which found that a high level of VP35 interferes with EBOV nucleocapsid formation in a VP35/NP overexpression system (51).

Due to the relatively low signal of VP35 in STAU1 Flag IP samples, we performed a reverse co-IP to confirm the interaction between these proteins. 293T cells were transfected with a plasmid expressing HA-VP35, and anti-HA IPs were performed in the presence or absence of RNase treatment. We observed endogenous STAU1 to co-IP with HA-VP35 (Fig. 5B). Additional co-IP experiments to map the interaction between STAU1 and VP35 suggested that both the C-terminal domain (containing amino acids 1 to 218) and the N-terminal domain (containing amino acids 219 to 340) of VP35 protein contribute to its interactions with STAU1 (Fig. S6A). In addition, we found that STAU1 coprecipitates with the EBOV VP30 transcription factor (Fig. S6B) in an RNA-independent manner, further supporting a role for STAU1 in EBOV RNA synthesis.

10.1128/mBio.01771-18.6 FIG S6 Interaction of STAU1 with EBOV VP35 and VP30. (A) 293T cells were transfected with equal amount of HA-tagged wild-type VP35 (VP35wt-HA), N-terminal domain of VP35 (VP35-N), or C-terminal domain of VP35 (VP35-C) expression plasmids. Forty-eight hours after transfection, cell lysates were harvested for the HA-IP experiments. Representative Western blot analysis from three independent HA-IP experiments is shown. (B) Coimmunoprecipitation of STAU1 with VP30-Flag. 293T cells were transfected with vector or VP30-Flag, and lysates with or without RNase treatment were harvested for IP using Flag antibody. The Western blot shows input and IP samples probed with Flag, STAU1, and PKR antibodies. Representative Western blot analysis from two independent experiments is shown. Download FIG S6, TIF file, 49.6 MB. Copyright © 2018 Fang et al. 2018 Fang et al. This content is distributed under the terms of the Creative Commons Attribution 4.0 International license.

We also probed for two cellular proteins that have been previously linked to VP35 and/or STAU1. In contrast to a previous study (52), we did not observe G3BP stress granule assembly factor 1 (G3BP1) to co-IP with VP35 (Fig. 5B). In contrast, we observed HA-VP35 to co-IP the eukaryotic initiation factor 2 (eIF2α) kinase 2 (EIF2AK2)/protein kinase R (PKR), which has previously been reported to interact with STAU1 (46) and be inhibited by VP35 (53, 54). We also observed PKR to co-IP with VP30 in an RNA-independent manner (Fig. S6B).

Together, our results show that STAU1 is able to interact with both VP35 and NP. Although each of these three proteins is capable of RNA binding, co-IP was resistant to RNase treatment, suggesting that RNA was not required for the interaction. Importantly, associations between STAU1 and either VP35 or NP were not altered when both proteins are coexpressed, which has been shown to result in formation of NP/VP35 helical structures that contain cellular RNA and resemble viral nucelocapsids (51). This result implies that STAU1 interaction with VP35 is not competed by NP and vice versa. Thus, we posit that these proteins can form a trimeric complex in cells.

**FIG 6:**
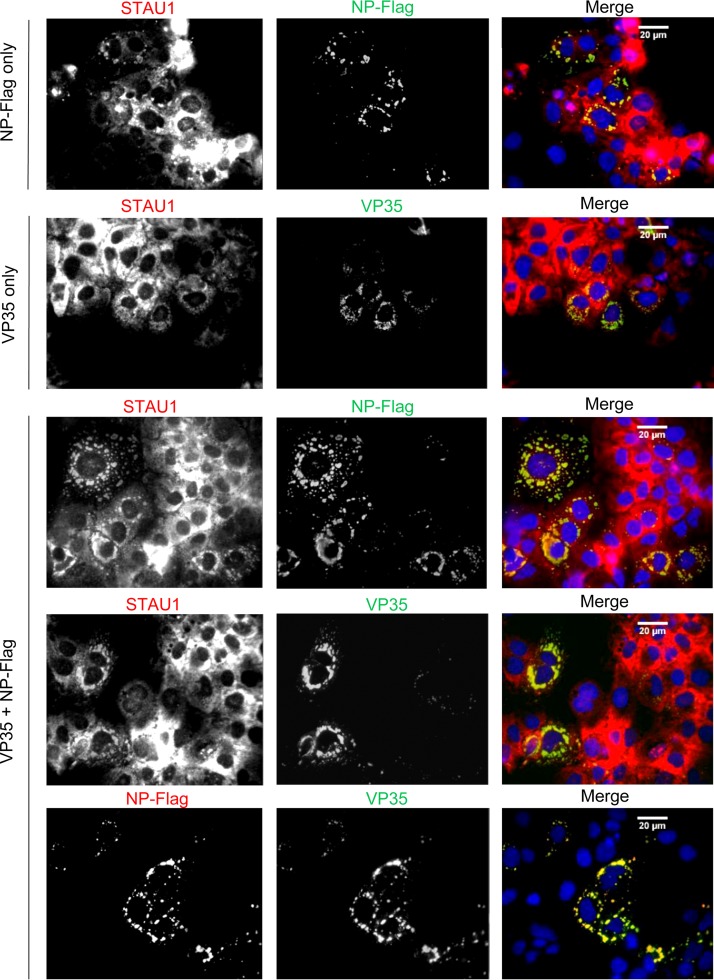
STAU1 redistributes to cytoplasmic inclusion bodies induced by expression of EBOV RNP components. HuH-7 cells were transiently transfected with NP-Flag or VP35 or cotransfected with plasmids expressing both proteins (at a ratio of 1:0.25 NP to VP35). At 24 h posttransfection, the cells were fixed and immunostained with anti-STAU1, anti-VP35, anti-Flag, or anti-NP antibodies for microscopy. Nuclei were visualized by Hoechst staining. Representative fields are shown from two independent experiments.

We further investigated the potential for colocalization of endogenous STAU1 and EBOV VP35/NP by immunofluorescence analysis. Specifically, we asked whether expression of NP, VP35, or both proteins is sufficient to recruit endogenous STAU1 to cytosolic granular structures formed by these proteins. First, consistent with previous studies (51, 55, 56), we found that transient NP expression alone was able to induce cytoplasmic inclusion bodies in HuH-7 cells (Fig. 6, NP-Flag only). Second, endogenous STAU1 was recruited to NP-induced cytoplasmic inclusion bodies (Fig. 6, NP-Flag only), consistent with our previous observation showing that, in EBOV-infected cells, endogenous STAU1 is localized in viral inclusion bodies containing VP35 (Fig. 3). In contrast to NP, we found that transient VP35 expression alone did not form structures reminiscent of NP-induced inclusion bodies but localized in smaller, more diffuse aggregates. However, endogenous STAU1 was still recruited to VP35 aggregates (Fig. 6, VP35 only). Finally, we found that coexpression of NP and VP35 led to formation of NP-induced inclusion bodies, which also contained STAU1 (Fig. 6, VP35 plus NP-Flag). Together with co-IP data shown in Fig. 5, these data suggest that EBOV VP35-NP complex recruits host STAU1 into cytoplasmic inclusion bodies that resemble EBOV inclusion bodies.

### STAU1 and EBOV NP/VP35/genomic RNA cofractionate in a density gradient.

Our results imply a possible ternary complex between STAU1, NP, and VP35 proteins. In addition, on the basis of our RAC data, we hypothesized that EBOV minigenome RNA, which harbors 3′ and 5′ extracistronic regions of the EBOV genome, would cofractionate with STAU1 together with NP and VP35 in sucrose density gradient centrifugation. To investigate the possibility that STAU1 can form a complex with EBOV RNP components, we cotransfected 293T cells with plasmids expressing EBOV NP, VP35, and the T7 promoter-driven EBOV minigenome with or without T7 polymerase expression plasmid. After 48 h, cytoplasmic lysates were harvested and separated on 20 to 60% sucrose density gradients. We fractionated the gradients and analyzed the distribution of target proteins and RNA in gradient fractions.

**FIG 7:**
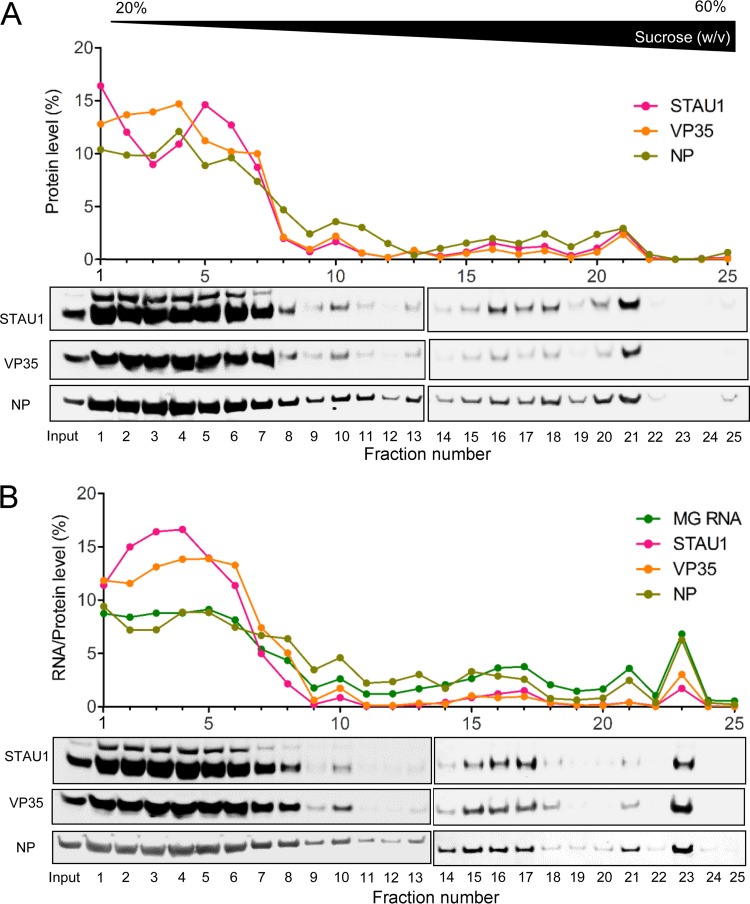
STAU1 and EBOV RNP components, NP, VP35, and EBOV minigenome (MG) RNA, cofractionate in sucrose density gradients. 293T cells were cotransfected with plasmids encoding NP, VP35, and EBOV minigenome plasmid with (A) or without (B) a T7 RNA polymerase expression plasmid. Forty-eight hours posttransfection, cell lysates were separated on a 20 to 60% (wt/vol) sucrose gradient. Twenty-five fractions were collected and analyzed for protein and RNA levels by Western blotting and RT-qPCR, respectively. Relative quantities of protein and RNA in each fraction were normalized against the total quantity in all fractions. See also Fig. S7 in the supplemental material.

Consistent with our previous findings, STAU1 primarily cosedimented with NP and VP35 in low-density fractions (Fig. 7A and Fig. S7A, fractions 1 to 8), but cosedimentation was also evident in the high-density fractions in the absence of EBOV minigenome RNA (Fig. 7A and Fig. S7A, fraction 21). In a repeat experiment, we also found PKR cosedimented with STAU1, NP, and VP35 in low-density fractions, both with and without EBOV minigenome RNA (Fig. S7). Importantly, we observed that expression of EBOV minigenome RNA led to a faster-migrating complex containing STAU1, NP, VP35, and EBOV minigenome RNA (Fig. 7B, fraction 23). In a repeat experiment, we observed a similar shift in STAU1/NP/VP35 due to expression of EBOV minigenome RNA (Fig. S7A and B). This result suggests that STAU1 is capable of forming an RNP complex with EBOV genomic RNA, NP, and VP35 proteins in cells (Fig. S8).

10.1128/mBio.01771-18.7 FIG S7 Repeat experiment showing STAU1 and EBOV RNP components (NP, VP35, and EBOV MG RNA) cofractionate in sucrose gradient. 293T cells were cotransfected with plasmids encoding NP, VP35, and EBOV minigenome plasmid with (A) and without (B) a T7 RNA polymerase expression plasmid. Forty-eight hours posttransfection, cell lysates were separated in a 20 to 60% sucrose gradient. Twenty-four fractions were collected and analyzed for protein and RNA levels by Western blotting and qRT-PCR, respectively. Relative quantities of protein and RNA in each fraction were normalized against the total quantity in all fractions. Download FIG S7, TIF file, 91.5 MB. Copyright © 2018 Fang et al. 2018 Fang et al. This content is distributed under the terms of the Creative Commons Attribution 4.0 International license.

10.1128/mBio.01771-18.8 FIG S8 Depiction of model showing hypothetical interactions between STAU1 and EBOV proteins at the 3′ and 5′ extracistronic regions of the viral genome. Download FIG S8, TIF file, 0.4 MB. Copyright © 2018 Fang et al. 2018 Fang et al. This content is distributed under the terms of the Creative Commons Attribution 4.0 International license.

## DISCUSSION

Our data provide evidence that STAU1 is the first cellular factor reported to associate with three essential components of the EBOV RNA synthesis machinery: NP, VP35, and VP30. The NP-VP35 complex serves as a critical backbone for the viral polymerase L to recognize NP-encapsidated RNA genomes, which is the prerequisite for both EBOV transcription and genome replication. Similarly, VP30 is also believed to bridge interactions between L and NP, and it is critical for transcription of viral mRNA (13, 57). Our observations further imply an association of STAU1 with the EBOV RNA genome-NP-VP35 complex, suggesting that regions of the EBOV genome are available for interaction with *trans*-acting factors in the presence of NP. Related to our finding that STAU1 preferentially binds to 3′ and 5′ extracistronic regions of the EBOV genome *in vitro*, we propose a model in which STAU1 is involved in the early formation of EBOV RNA synthesis machinery on the 3′ extracistronic region of the EBOV genome, and likewise, in the termination and release of EBOV RNA synthesis machinery on the 5′ extracistronic region (see Fig. S8 in the supplemental material).

Previous efforts have been made to understand EBOV host factor biology by focusing on protein-protein interactions between viral and host proteins (58–62). To provide more insights into how host factors regulate EBOV replication through *cis*-acting elements in EBOV RNA, we performed a RAC-MS screen and discovered 14 host RBPs specifically enriched for the selected EBOV noncoding RNAs. Among these RBPs, ILF2, ILF3, HNRNPL, and STAU1 stand out as strong EBOV candidate host factors because all three siRNAs tested caused a significant reduction of EBOV infection. While HNRNPL preferentially associated with the 5′ UTR of EBOV NP mRNA, ILF2, ILF3, and STAU1 selectively interacted with the EBOV trailer. It is noteworthy that ILF3 (also known as DRBP76/NF90) was previously identified as a VP35 interactor as well as an EBOV polymerase suppressor (61). Although this previous study did not observe a dramatic effect of reduced ILF3 expression on EBOV infection in 293T cells, the data we present here showed that depletion of ILF3 in HuH-7 cells significantly impaired EBOV infection. This discrepancy could be explained by cell line differences. Nevertheless, the identification of ILF2 (or NF45) in our screen, which is physically and functionally linked to ILF3 (44), highlights the important role that ILF3 perhaps has in EBOV infection.

We focused on STAU1 as a novel EBOV host factor that promotes efficient virus infection. STAU1 is a protein that binds to stable RNA secondary structure (63), a tubulin-binding protein that interacts with cytoskeleton (50), a ribosome-associated protein (64, 65) that accumulates in stress granules induced by certain types of stress (66), and a key mediator of mRNA decay (67, 68). These biochemical characteristics enable STAU1 to fulfill different biological functions: from controlling the localization to enhancing the translation of its target RNAs. Therefore, elucidation of a functional domain(s) of STAU1 that is responsible for EBOV host factor activity may allow generation of targeted inhibitors antagonizing the STAU1 proviral effect.

Previous studies of several RNA viruses have revealed that STAU1 binding to viral RNA regulates infection. For instance, STAU1 facilitates translation of hepatitis C virus RNA through binding to the internal ribosome entry site (46). STAU1 plays a role in the production of viral particles for both influenza A virus (47) and human immunodeficiency virus type 1 (48). Our RAC-MS results indicated that STAU1 preferentially associates with EBOV genomic RNA and not with the 5′ UTR of two viral mRNAs. Therefore, it is unlikely that STAU1 promotes EBOV mRNA translation; however, we tested only the 5′ UTRs of NP and VP24 transcripts, so we cannot eliminate the possibility that STAU1 may bind to other UTRs and participate in their translation. Another caveat is that the EBOV genomic RNA used in the RAC experiment was naked, while during infection, it is likely to be mostly encapsidated by NP and therefore relatively inaccessible (69). Nevertheless, the facts that STAU1 is recruited to sites of viral RNA synthesis and forms a complex in cells with NP, VP35, and minigenome RNA suggest interactions between STAU1 and EBOV genomic RNA in infected cells.

Several other stress granule (SG) markers (for instance, the initiation factors eIF3 and eIF4G) were previously found to localize on discrete, compact granules within EBOV inclusion bodies (30). In contrast to this, STAU1 in EBOV inclusion bodies appeared in a heterogeneous pattern that changed over the course of infection. In some areas near the border of EBOV inclusion bodies, where VP35 was almost absent, STAU1 formed small aggregates that branched out of the inclusion border (Fig. S5C). This distinct distribution of STAU1 suggests that it may function differently than other SG proteins with regard to interactions with EBOV inclusion bodies.

Given the correlation between the level of STAU1 expression and EBOV minigenome activity, it is of interest to pinpoint the molecular event(s) STAU1 regulates during EBOV RNA synthesis. For nonsegmented, negative-sense RNA viruses, the first step of viral RNA synthesis is primary transcription in order to generate sufficient levels of viral proteins. When a supply of NP has been synthesized, genome replication and secondary transcription are allowed to commence, leading to the production of encapsidated antigenomes and progeny genomes as well as naked mRNAs (70). Attempts to distinguish whether STAU1 enhances genome replication or transcription using the minigenome system by strand-specific quantitative PCR (qPCR) were not successful, however, due to residual DNA from transfected plasmids despite DNase treatment of RNA samples. Future studies using other model systems (i.e., replication-deficient minigenome) are required to ascertain whether STAU1 participates in EBOV transcription (71).

Preliminary characterization of STAU1-associated EBOV minigenome RNP (reconstituted by EBOV minigenome RNA, NP, and VP35) revealed another interesting cellular player, PKR. Although PKR is well-known for its antiviral function by sensing viral RNA and phosphorylating the host translation initiation factor eIF2α (72), it has also been implicated in the phosphorylation and regulation of the HIV *trans*-acting protein Tat, which binds to the transactivation-responsive element (TAR) in the HIV genome, in the context of viral infection (73, 74). Interestingly, no host kinase has yet been reported to regulate phosphorylation of EBOV VP30, even though a growing number of studies indicate a crucial role of dynamic VP30 phosphorylation in EBOV RNA synthesis and RNP assembly (19, 21, 55, 75, 76). Although several reports indicate that EBOV antagonizes PKR activity (53, 54), one can imagine scenarios in which PKR is hijacked by this STAU1-containing viral RNP complex to dynamically control the phosphorylation of EBOV VP30. Further investigation is needed to clarify the role of PKR in EBOV infection.

In conclusion, we identified STAU1 as the first host factor reported to interact with multiple EBOV RNP components, highlighting the significance of this RBP for the EBOV life cycle. These interactions together with redistribution of STAU1 to EBOV inclusion bodies and to NP-induced inclusion bodies link STAU1 to EBOV RNA synthesis, for which we speculate that STAU1 facilitates both the initiation and termination steps. It will be of interest to clarify the exact molecular events occurring during initiation and termination of EBOV RNA synthesis and how STAU1 may participate in these processes. Our study contributes to knowledge of the role of host factors in EBOV RNA synthesis and provides a novel cellular target for the development of possible therapeutic interventions to combat EBOV infection.

## MATERIALS AND METHODS

### Cell lines and viruses.

HuH-7 and 293T cells were maintained in Dulbecco’s modified Eagle medium supplemented with 10% fetal bovine serum (FBS), penicillin (100 U/ml), streptomycin (100 μg/ml), nonessential amino acids, and plasmocin (2.5 μg/ml). Vero-E6 cells were maintained in minimum essential medium supplemented with 10% FBS, 1% sodium pyruvate, and 0.1% gentamicin sulfate. All cells were grown at 37°C and 5% CO_2_. EBOV strain Mayinga and Mayinga expressing green fluorescent protein (GFP) (45) were propagated in Vero-E6 cells, and titers were determined by plaque assay (limit of detection, 100 PFU). Work with EBOV was done under biosafety level 4 (BSL-4) conditions at the Galveston National Laboratory. Virus inactivation was performed according to standard operating procedures (SOP).

### Plasmids, antibodies, and siRNAs.

The pCI-neo-STAU155R-FLAG plasmid was a gift from Lynne Maquat (University of Rochester). Plasmids pCEZ-NP, pCEZ-VP35, pCEZ-VP30, pCEZ-L, and pCAGGS-T7 were kindly provided by Yoshihiro Kawaoka (University of Wisconsin). The EBOV minigenome system was kindly provided by Elke Mühlberger (Boston University). The pcDNA5-eVP35-HA plasmid and deletion variants were constructed by introducing a C-terminal hemagglutinin (HA) epitope tag by PCR amplification and cloning into pcDNA5/FRT/TO using KpnI and NotI. The pRL-TK plasmid was purchased from Promega. The pCAGGS-eNP-flag plasmid was a gift from Daniel Engel (University of Virginia).

Antibody 6C5 for VP35 was purchased from Kerafast. Anti-NP antibody was purchased from IBT Bioservices. Anti-STAU1 (EPR7966) and anti-HA tag (16B12) antibodies were purchased from Abcam. Antitubulin antibody (B-5-1-2) was purchased from Invitrogen. All small interfering RNAs (siRNAs) were purchased from Qiagen (see Table S1 in the supplemental material), including the AllStars negative control siRNA). Band intensity quantification for Western blots was performed using Image Studio version 5.2 (Li-Cor).

10.1128/mBio.01771-18.9 TABLE S1 siRNAs used in this study. Detailed information on the siRNAs in Fig. 1C is shown. Download Table S1, DOCX file, 0.0 MB. Copyright © 2018 Fang et al. 2018 Fang et al. This content is distributed under the terms of the Creative Commons Attribution 4.0 International license.

### Identification of EBOV RBPs by RAC-MS.

RNA affinity chromatography combined with mass spectrometry (RAC-MS) experiments were performed as described previously (77). Briefly, HuH-7 cytoplasmic lysate containing 5 mg of total protein was used for each binding reaction mixture with 25 pmol of noncapped, *in vitro*-transcribed RNA immobilized on tobramycin-coupled *N*-hydroxysuccinimide (NHS)-activated Sepharose matrices. Eluted proteins were subjected to either quantitative mass spectrometry or Western blot analysis. For proteins identified by MS, the abundance of each protein is indicated by normalized total spectral counts. We calculated the average abundance of identified proteins in eluate from each RNA construct and normalized it to that from the matrix, which we termed RNA/matrix ratio. From duplicate RAC-MS experiments, we classified host RNA-binding proteins (RBPs) in each sample as proteins with RNA/matrix ratios of >2. To identify RBPs preferentially bound to EBOV RNA, we introduced two enrichment factor criteria to exclude proteins bound nonspecifically to all RNAs. We defined enrichment factor 1 as the ratio of the protein abundance in eluate from a particular EBOV RNA to that from the DDX39B reference RNA. Similarly, we defined enrichment factor 2 as the ratio of the protein abundance in eluate from a particular EBOV RNA to the mean of all RNAs tested. Proteins specifically bound to EBOV RNA were selected with at least one enrichment factor larger than 2.

### siRNA screening.

HuH-7 cells (5 × 10^3^) were seeded into 96-well plates and transfected the next day with 10 nM siRNA using Lipofectamine RNAiMAX. Cells were transferred to BSL-4 and 48 h later were infected with EBOV expressing enhanced GFP (EBOV-eGFP) at an MOI of 1 PFU per cell. At 72 h postinfection (hpi), the cells were fixed with formalin and removed from BSL-4 conditions. Nuclei were visualized by Hoechst staining. Rates of infected cells were determined by measuring GFP-positive cells using an Opera Phenix high-content imager. Representative images of fixed and stained monolayers were taken using a wide-field fluorescence microscope (Olympus).

### EBOV growth kinetics.

HuH-7 cells (3 × 10^4^) were seeded in 24-well plates and transfected with siRNA the next day as described above. Twenty-four hours later, cells were maintained under BSL-2 conditions for Western blot analysis or transferred into BSL-4 facilities for infection. Forty-eight hours after transfection, cells were infected with EBOV-eGFP at an MOI of 3 or 0.01 for 1 h. Infection of the cells was followed by removal of the inoculum, washing the cells with phosphate-buffered saline (PBS) three times, and adding fresh medium containing 2% FBS to the cells. Aliquots of supernatant were harvested every 24 h for virus titration.

### EBOV minigenome assay.

293T cells (3 × 10^4^) were seeded in 24-well plates and transfected the next day with siRNA at a final concentration of 10 nM per well using Lipofectamine RNAiMAX 48 h prior to minigenome transfection. For STAU1 overexpression, 6 × 10^4^ 293T cells were seeded in 24-well plates and transfected the next day with 500 ng of pCI-neo-STAU1^55R^-FLAG or 500 ng of a control pCI plasmid using Lipofectamine 2000 24 h prior to minigenome transfection. Experiments involving the EBOV minigenome were performed essentially as previously described (19). Confluent monolayers were transfected with the following plasmids: 50 ng pCEZ-NP, 50 ng pCEZ-VP35, 30 ng pCEZ-VP30,400 ng pCEZ-L, 100 ng pCAGGS-T7, 100 ng EBOV minigenome, and 4 ng pRL-TK. A control was included in every assay in which the pCEZ-L plasmid was omitted to verify active RNA synthesis. Plasmids were transfected with TransIT-LT1 transfection reagent (3 µl/µg plasmid). At 48 h posttransfection, cells were lysed and subjected to the dual-luciferase reporter assay (Promega). Knockdown of STAU1 was consistent between the time of minigenome plasmid transfection (Fig. 4B) and harvesting of lysates for luciferase assay (unpublished data).

### Immunoprecipitation and RNA-IP.

293T cells (6 × 10^5^) seeded in 100-mm dishes were transfected the next day with 14 μg total plasmid(s) using Lipofectamine 2000. Forty-eight hours posttransfection, cells were lysed in radioimmunoprecipitation assay (RIPA) buffer containing protease inhibitors. Anti-HA or anti-Flag M2 agarose beads were blocked in NT2 buffer (50 mM Tris-HCl [pH 7.4], 150 mM NaCl, 1 mM MgCl_2_, and 0.05% NP-40) with bovine serum albumin (BSA) (0.5 mg/ml) for 30 min. One milligram of cell lysate with or without RNase treatment (0.4 U/ml RNase A and 16.6 U/ml RNase T1, 37°C, 15 min; verified to degrade total RNA in 1 mg of 293T lysate) was added to agarose beads and incubated overnight at 4°C with rotation. After four washes with NT2 buffer, proteins were eluted in sample buffer and analyzed by Western blotting. In RNA immunoprecipitation (RNA-IP) experiments, RNA was eluted from IP reaction mixtures using TRIzol-LS and analyzed by reverse transcription-quantitative PCR (RT-qPCR) using primers that specifically recognize EBOV trailer.

### Immunofluorescence analysis (IFA).

HuH-7 cells grown on 8-well chamber slides were fixed with 4% paraformaldehyde (PFA) for 15 mins, permeabilized with 0.5% Triton X-100 for 10 min, and incubated in blocking solution (1% normal goat serum in PBS) for 1 h at room temperature (RT). Primary antibodies were incubated for 2 h at RT or overnight at 4°C. After the cells were washed with PBST (PBS containing 0.1% Tween 20), secondary antibodies and Hoechst stain were added, and cells were incubated for 1 h at RT followed by washes with PBST. Monolayers were fixed by formalin and removed from BSL-4 following SOP. To neutralize formalin, monolayers were treated with 0.1 M glycine for 10 min at room temperature, mounted in ProLong Gold antifade reagent and analyzed by laser scanning confocal microscopy using an Olympus FV1000 confocal microscope with a 60× immersion oil lens. The number and area of EBOV inclusion bodies were quantified using Image J software.

### IFA of cells expressing EBOV proteins.

HuH-7 cells (3 × 10^5^) grown on six-well glass bottom plates were transfected with a total of 2.5 μg of plasmid(s) in different combinations: NP-flag only, VP35 only, and NP-flag and VP35 (4:1). At 24 h posttransfection, cells were fixed, permeabilized, and incubated with different combinations of antibodies. After the final wash with PBST, cells were mounted and analyzed by wide-field fluorescence microscopy (Olympus) using an Olympus IX71 microscope with a 60× immersion oil lens.

### Sucrose density gradient analysis.

Cell fractionation on sucrose gradients was adapted from previous studies (48, 65). 293T cells (1.6 × 10^7^) grown in 100-mm dishes were transfected with plasmids expressing EBOV NP (5.6 μg), VP35 (1.4 μg), and T7 polymerase (3.5 μg) with or without the T7 promoter-driven EBOV minigenome plasmid (3.5 μg). After 48 h, cells were harvested and homogenized in 2 ml lysis buffer (10 mM HEPES [pH 7.5], 12.5% sucrose, 1 mM EDTA, 1× protease inhibitor). Sequential centrifugations at 700 × *g* and 1,000 × *g* for 5 min were performed, and the resulting supernatants were layered onto continuous 20 to 60% sucrose gradients in 10 mM HEPES (pH 7.5) and 1 mM MgCl_2_ and centrifuged at 31,000 × *g* for 2.5 h in a SW40 rotor (Beckman). Fractions were collected from top to bottom, and protein and RNA were collected from each fraction for quantification by Western blotting and RT-qPCR, respectively.

### Statistical analysis.

Details regarding number of replicates and type of statistical test can be found in figure legends. Statistical analyses were performed using GraphPad Prism version 5.01.
