# Supplementary figures and images for: Staufen1 Interacts with Multiple Components of the Ebola Virus Ribonucleoprotein and Enhances Viral RNA Synthesis
Source: mBio. 2018 Oct 9;9(5):e01771-18. doi: 10.1128/mBio.01771-18 (PMC6178623; doi:10.1128/mBio.01771-18)

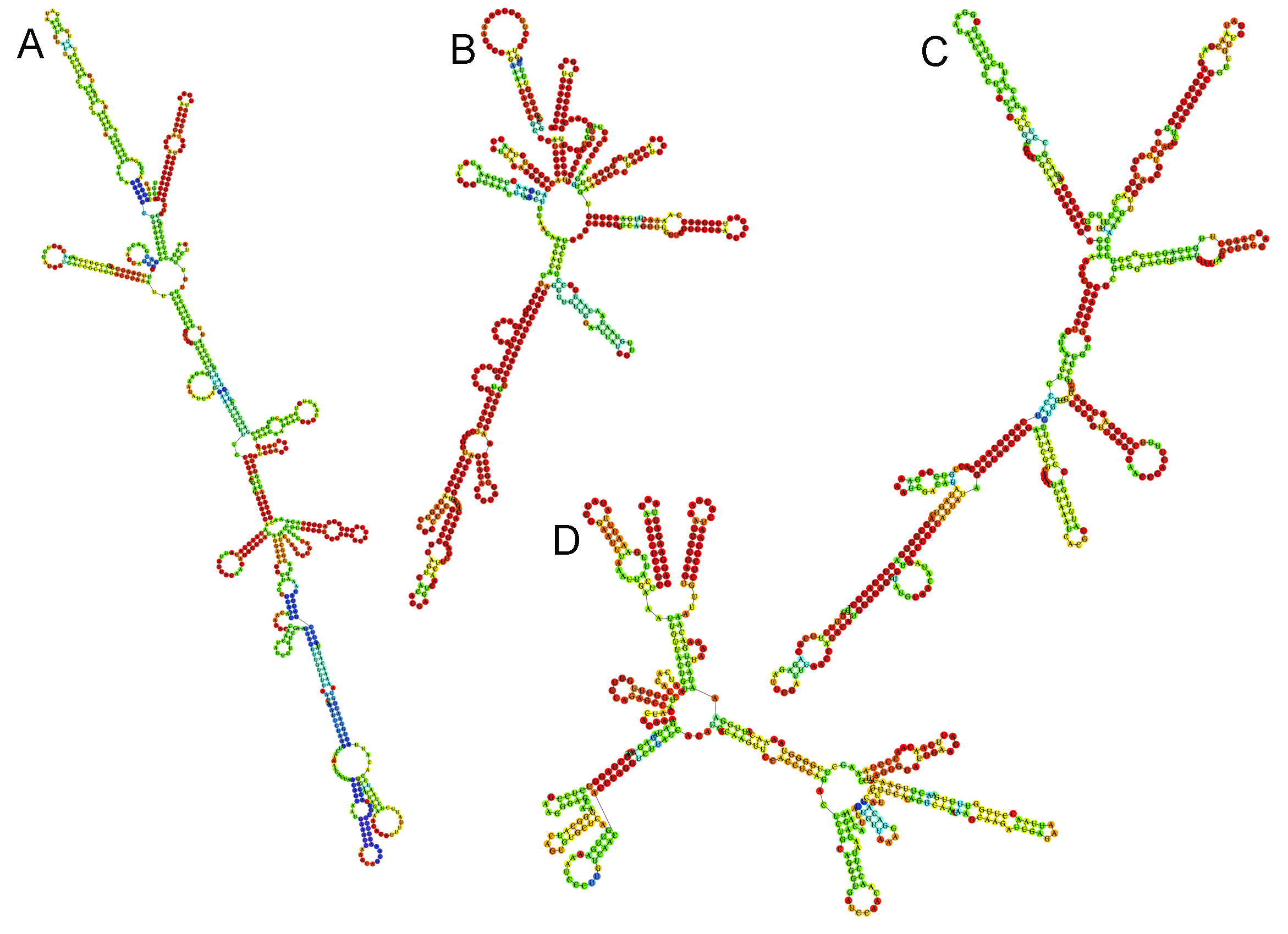

Supplement: FIG S1 [file mbo005184093sf1.tif]

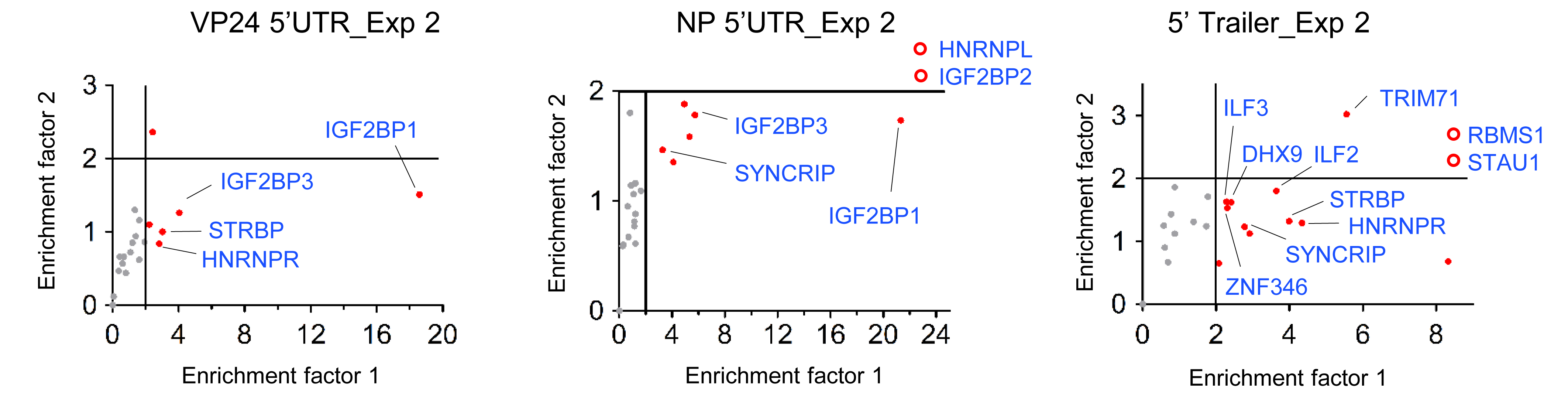

Supplement: FIG S2 [file mbo005184093sf2.tif]

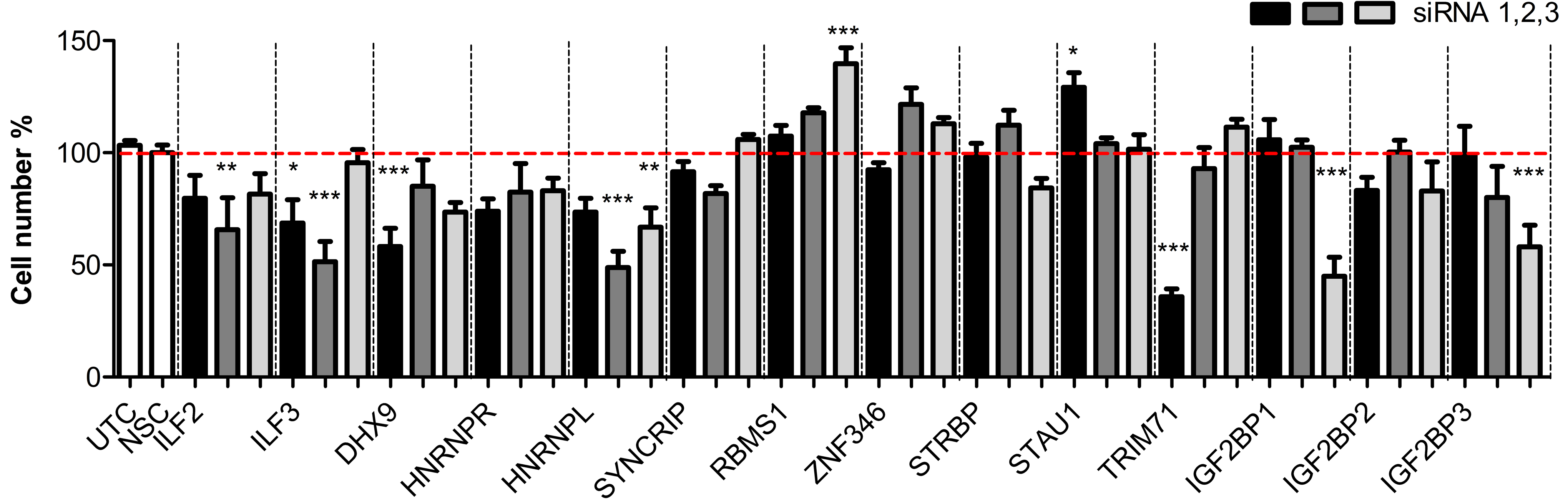

Supplement: FIG S3 [file mbo005184093sf3.tif]

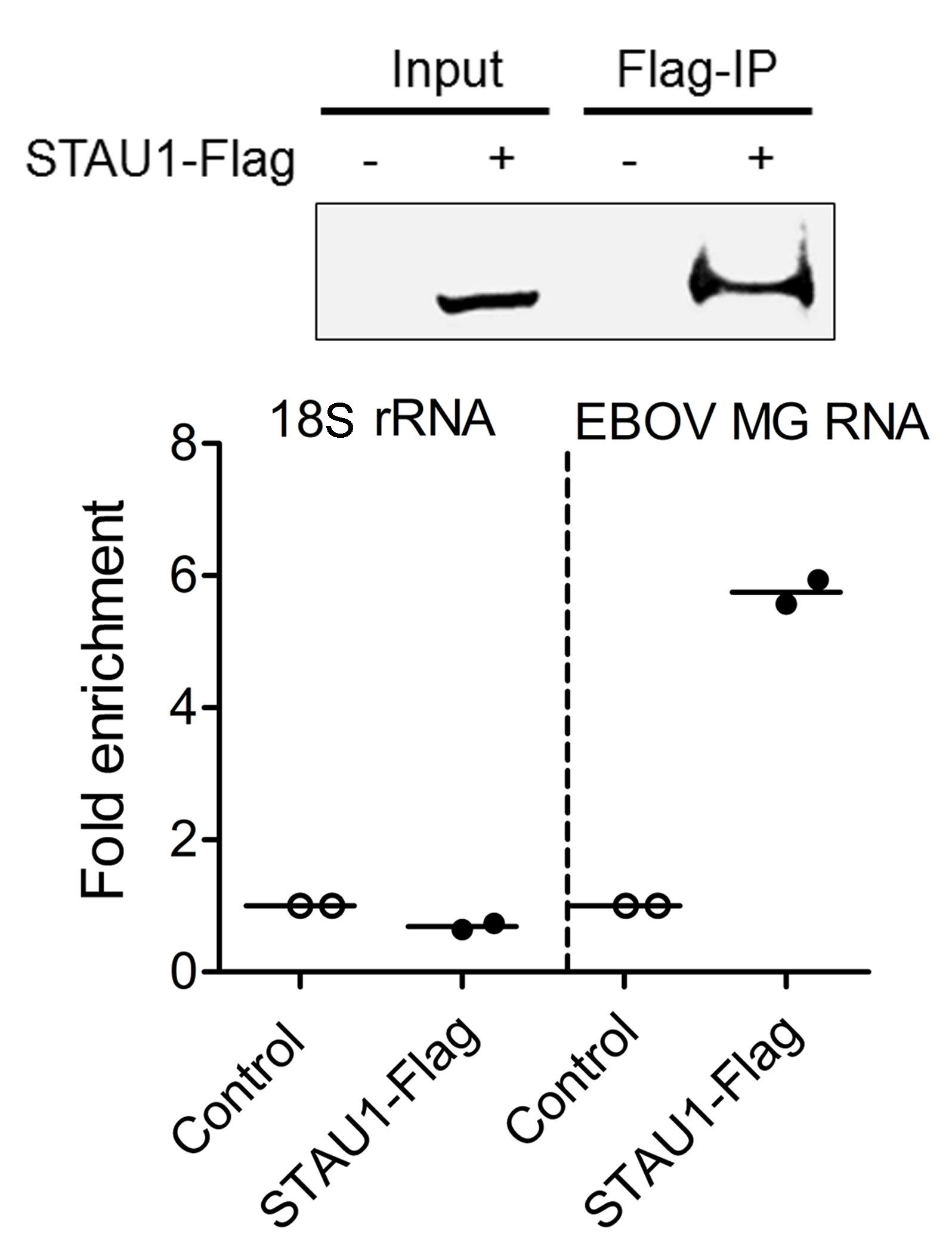

Supplement: FIG S4 [file mbo005184093sf4.tif]

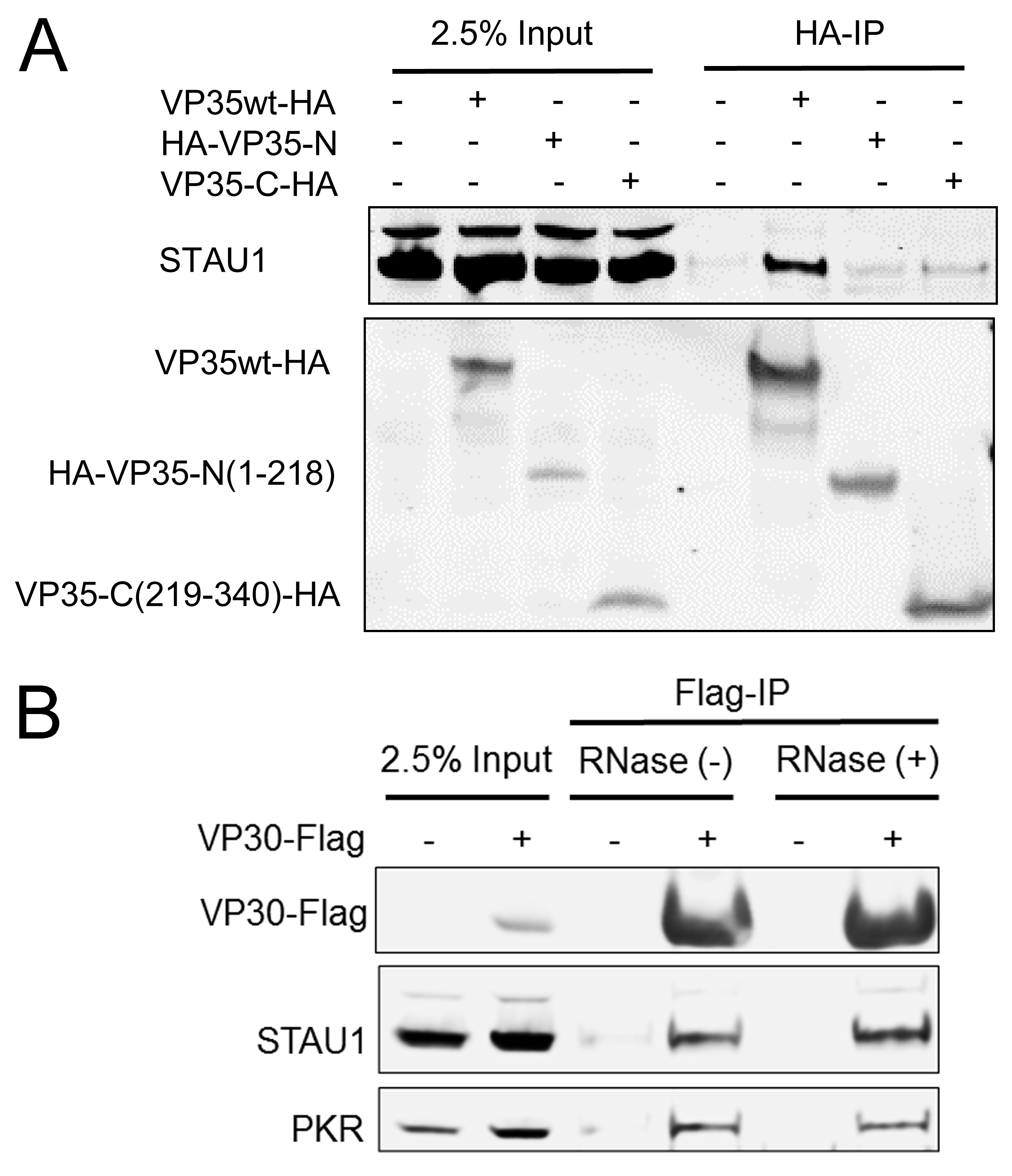

Supplement: FIG S6 [file mbo005184093sf6.tif]

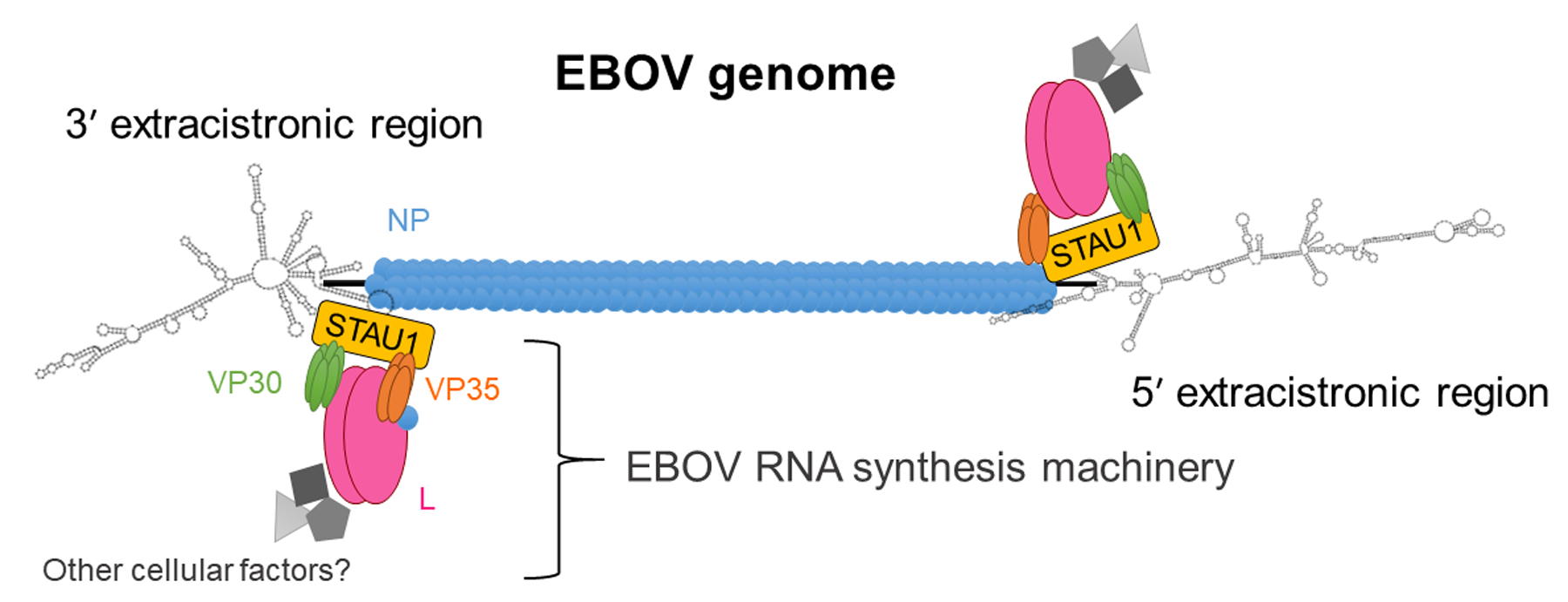

Supplement: FIG S8 [file mbo005184093sf8.tif]
