# Supplementary material for: Staufen1 Interacts with Multiple Components of the Ebola Virus Ribonucleoprotein and Enhances Viral RNA Synthesis
Source: mBio. 2018 Oct 9;9(5):e01771-18. doi: 10.1128/mBio.01771-18 (PMC6178623; doi:10.1128/mBio.01771-18)
Supplement: TABLE S1 [file mbo005184093st1.docx]

**Table S1. Related to Figure 1C. siRNAs used in this study.**

| siRNA Name | Catalog Number | Target Sequence |
| --- | --- | --- |
| ILF2_s1 | SI02664851 | CTCCATAGAAGTGTCATTCCA |
| ILF2_s2 | SI02664844 | CAGGCCCTTTGTACCACATAT |
| ILF2_s3 | SI04435452 | CACAGTCATGACCCTAGAACA |
| ILF3_s1 | SI02648779 | CAAGAGTTGAAGTATTGATAA |
| ILF3_s2 | SI04949455 | GTGGAGGTTGATGGCAATTCA |
| ILF3_s3 | SI02648786 | CAAGCTGCACGTGGCCGTTAA |
| DHX9_s1 | SI04327204 | CCAGAGCCTTTCATCAATGAA |
| DHX9_s2 | SI04186574 | CTGGGCTATATCCATCGAAAT |
| DHX9_s3 | SI04163516 | AGCGTTCGATTTGAGTCTATA |
| HNRNPR_s1 | SI00439943 | ACGGAGGTATTTGTAGGCAAA |
| HNRNPR_s2 | SI04161304 | CAGAGGGTTTGGTGGACGTTA |
| HNRNPR_s3 | SI04201260 | CACTGCGTATGAAGATTATTA |
| HNRNPL_s1 | SI04208323 | AACTACGATGACCCGCACAAA |
| HNRNPL_s2 | SI03208527 | CTGCGATGAGCTGGGAGTGAA |
| HNRNPL_s3 | SI00300475 | AAAGCCTACACGCTTGAATGT |
| SYNCRIP_s1 | SI05157222 | AAAGTAGCAGATTCTAGTAAA |
| SYNCRIP_s2 | SI00737352 | GAGGATGAACTTGTTCCATTA |
| SYNCRIP_s3 | SI04300604 | CGCGGTAGAGCCGGTTATTCA |
| RBMS1_s1 | SI04335184 | AAACTTCTATTACACCCTATA |
| RBMS1_s2 | SI04285407 | AACGTAGTGTGAAGTGTCTTA |
| RBMS1_s3 | SI04287164 | CTGGTGAAGCTCTGTCAACCA |
| ZNF346_s1 | SI00098854 | GGGCCTCATGAAGGTCTTTAA |
| ZNF346_s2 | SI03029306 | AACGACCCTGTCATGGCTCAA |
| ZNF346_s3 | SI03032183 | AAGATAGTGCTGAACTCCATA |
| STRBP_s1 | SI04263959 | AATGCGGCAAATAATAAGAAA |
| STRBP_s2 | SI04195625 | GAGGATTGGCCTGGTTGCAAA |
| STRBP_s3 | SI04184411 | CAGGCAAGGGCAAATGGATTA |
| STAU1_s1 | SI04231584 | CTCGGATGCAGTCCACCTATA |
| STAU1_s2 | SI04269335 | CTGCGTGTGGTCCGTATGGAA |
| STAU1_s3 | SI04272387 | CTGCCTGCAGTTGAACGAGTA |
| TRIM71_s1 | SI03186477 | CCCGTGTGCGACCAGAAAGTA |
| TRIM71_s2 | SI03238690 | TGGGACATACGTGGTGAGTTA |
| TRIM71_s3 | SI04655609 | CTGTGCAAGTTTGGTGCTCAA |
| IGF2BP1_s1 | SI02655394 | CACATTTAATTCCTGGATTAA |
| IGF2BP1_s2 | SI04314898 | CTGGGCGATGAAGGCCATCGA |
| IGF2BP1_s3 | SI04360559 | AAGGTTTAGTGGGATGAAGGA |
| IGF2BP2_s1 | SI03176593 | CAGGGCGTTAAATTCACAGAT |
| IGF2BP2_s2 | SI04138820 | CAGCGAAAGGATGGTCATCAT |
| IGF2BP2_s3 | SI04367020 | CCCGGGTAGATATCCATAGAA |
| IGF2BP3_s1 | SI04316165 | CTGGTACTAGCTAAGAAATAA |
| IGF2BP3_s2 | SI03230759 | TCCAGATACCTTGACGCAGAA |
| IGF2BP3_s3 | SI04234167 | CAGCAATCAGCTAACAGGCAA |

In addition to siRNAs listed above, the AllStars Neg. Control siRNA (Cat# SI03650318) was used as a non-silencing control (NSC). All siRNAs were purchased from Qiagen.
